# Glutathione-Stabilized Copper Nanoclusters as a Switch-Off Fluorescent Sensor for Sensing of Quercetin in Tea Samples

**DOI:** 10.3390/foods14152750

**Published:** 2025-08-06

**Authors:** Xueqing Gao, Xuming Zhuang

**Affiliations:** 1 School of Computer Science and Technology, Shandong Technology and Business University, Yantai 264005, China; 2 School of Chemistry and Chemical Engineering, Yantai University, Yantai 264005, China; xmzhuang@iccas.ac.cn

**Keywords:** copper nanoclusters, fluorescence, switch-off sensor, quercetin

## Abstract

Quercetin, a natural polyphenolic flavonoid with antioxidant and anti-allergic properties, is extensively found in foods and holds significant importance for human health. In this study, a simple switch-off fluorescent sensor based on copper nanoclusters (Cu NCs) was proposed for the sensitive determination of quercetin. Glutathione acted as the reducing and protective agent in the synthesized process of Cu NCs via a facile, green one-pot method. As anticipated, the glutathione-capped Cu NCs (GSH-Cu NCs) exhibited favorable water solubility and ultrasmall size. The fluorescence property of GSH-Cu NCs was further enhanced with Al^3+^ ion through the aggregation-induced emission effect. When quercetin was present in the sample solution, the system exhibited effective fluorescence quenching, which was attributed to the internal filter effect. The GSH-Cu NCs/Al^3+^-based fluorescent sensor showed a good linear relationship to quercetin in the concentration range from 0.1 to 60 μM. A detection limit of 24 nM was obtained. Moreover, the constructed sensor was employed for the successful determination of quercetin in tea samples.

## 1. Introduction

Quercetin is a bioactive flavonoid, which is naturally distributed in plants, fruits, vegetables and drinks. Recently, the significant value of quercetin in many traditional Chinese medicines has also attracted considerable attention from researchers [1,2]. Due to its remarkable pharmacological properties, including antioxidant, antibacterial, anti-inflammatory, anticancer and hypoallergenic effects, quercetin has been used in the treatment of cardiovascular disease, neurodegenerative disease and cancer [3,4,5]. Additionally, it is reported that quercetin is beneficial for delaying aging [6]. However, excessive intake of quercetin may cause liver stress and disrupt the expression of key transcription factors or proteins, leading to alterations in metabolic properties [7]. Therefore, it is necessary to established a simple and reliable approach for the accurate determination of quercetin.

Currently, the developed techniques for quercetin detection include capillary electrophoresis, high-performance liquid chromatography, electrochemical method, colorimetry, fluorometry and so on [8,9,10,11,12]. Among them, fluorometry has gained tremendous attention because of its inherent characteristics of simple operation, rapid response, low cost and good sensitivity. For example, Zhang and co-workers prepared the Mn-doped ZnS quantum dots (QDs) and used for bioflavonoid quercetin detection in human urine and human serum [13]. Rajamanikandan and co-workers designed a fluorescent probe based on Ti_3_C_2_ MXene QDs for the quantification of quercetin in food samples such as orange [14]. Kadian and Manik fabricated the fluorescent sulfur doped graphene QDs for effective sensing of quercetin in several red wine [15]. Nevertheless, the potential ecotoxicological risk of fluorescent QDs limited their further applications. Based on that, the development of biocompatible and eco-friendly fluorescent sensor is essential for the determination of quercetin.

Metal nanoclusters (MNCs), a class of novel fluorescent nanomaterials composed of a few atoms, possess molecule-like properties due to the size of them that is close to the Fermi wavelength of electrons. Compared to metal nanoparticles, the levels of discrete energy induced by quantum confinement in MNCs allow them possessing superior optical properties. Coupling the features of biocompatibility and environmental friendliness, MNCs have been successfully employed for catalysis, bioimaging, drug delivery, chemical and biological sensing [16,17,18,19]. Compared with gold and silver nanoclusters, the lower cost and easier availability endow copper nanoclusters (Cu NCs) with the wider application prospect [20]. For example, Sam et al. reported a tannic acid-capped Cu NCs-based fluorescent probe for the cost-effective determination of hemoglobin [21]. Guo and co-workers proposed a facile fluorescence strategy for sensing of baicalein using Cu NCs synthesized with trypsin as the template [22]. Chen et al. prepared fluorescent Cu NCs using double-stranded DNA as the template for analyzing of β-glucosidase activity [23]. While the copper ion can be toxic in certain contexts, the Cu NCs stabilized by biomolecules, such as tannic acid and trypsin, can effectively reduce copper ion leaching and minimize associated risks. In particular, compared to larger nanoparticles, the ultrasmall size and surface passivation of Cu NCs further limit their cytotoxic effects. Thus, Cu NCs demonstrate a great promise as one of the most promising candidates for fluorescent bioassays.

**Figure 1:**
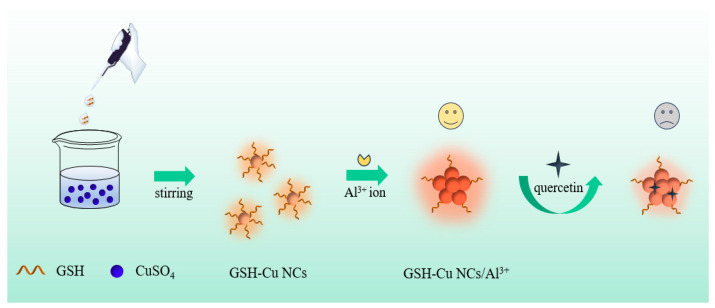
Schematic diagram of the synthesis of the GSH-Cu NCs/Al^3+^ and fluorescence quenching concept of quercetin.

Herein, we prepared water-dispersible Cu NCs with glutathione (GSH) molecules as the reducing and protective agent via a facile one-pot method, and labeled as GSH-Cu NCs. Subsequently, Al^3+^ ion was added to enhance the fluorescence of free Cu NCs through aggregation-induced emission effect. The formed GSH-Cu NCs/Al^3+^ were characterized by different techniques including the transmission electron microscopy (TEM), energy dispersive spectrometer (EDS), X-ray photoelectron spectroscopy (XPS), Fourier transform infrared spectrometer (FT-IR), and optical spectroscopy. When quercetin was added to the system, as depicted in Figure 1, the orange-red fluorescence of the GSH-Cu NCs/Al^3+^ could be effectively quenched. This phenomenon demonstrated the as-synthesized GSH-Cu NCs/Al^3+^ functions as a sensitive switch-off fluorescent sensor for quercetin detection.

## 2. Materials and Methods

### 2.1. Reagents and Instruments

CuSO_4_·5H_2_O and glucose (Glu) were procured from Sinopharm Chemical Reagent Co., Ltd., China. Quercetin, L-glutathione (GSH), ascorbic acid (AA), cysteine (Cys), uric acid (UA), and dopamine (DA) were acquired from Sigma-Aldrich. Glycine (Gly), proline (Pro) and histidine (His) were sourced from Aladdin Chemistry Co., Ltd., China. Catechin hydrate (Cat) was purchased from Macklin Biochemical Co., Ltd. (Tianjin, China). All other involved chemicals were obtained from Sinopharm Chemical Reagent Co., Ltd. (Shanghai, China). Commercial green tea, black tea and oolong tea samples were gained from a local supermarket in Yantai, China. Deionized water was collected via a Milli-Q system.

The microscopic morphology of the nanomaterials was characterized by a JEM-2010 transmission electron microscope (JEOL, Tokyo, Japan). The changes in the elementary composition of the nanomaterials were characterized by EDS (JEOL-7900F, Tokyo, Japan) and XPS (Thermo ESCALAB 250XI, Waltham, MA, USA) techniques. A Nicolet 5700 Fourier transform infrared spectrometer (Thermo Fisher Scientific, Waltham, MA, USA) was employed to analyze the structural variation of the nanomaterials. The fluorescence and absorption spectra of the nanomaterials were recorded by a Cary Eclipse fluorescence spectrophotometer (Varian, Palo Alto, CA, USA) and a 2450 UV-visible spectrophotometer (Shimadzu, Kyoto, Japan), respectively.

### 2.2. Synthesis of GSH-Cu NCs/Al^3+^

The fabrication of GSH-Cu NCs/Al^3+^ was conducted with a facile method described in previous work [24]. Typically, 2.5 mL of pre-prepared GSH aqueous solution (50 mM,) was gradually added into the same volume of CuSO_4_ solution (10 mM). Then, the pH of the mixed solution was adjusted to 6.0 using fresh NaOH (1.0 M) with continuous stirring. During this process, the appearance of the mixture would change from muddy to transparent, indicating the successful formation of GSH-stabilized Cu NCs. Following the addition of AlCl_3_ solution (10 mM, 50 μL) into the as-synthesized GSH-Cu NCs, and the resulting solution reverted to a turbid state. Finally, the GSH-Cu NCs/Al^3+^ were preserved under the condition of 4 °C for subsequent measurements.

### 2.3. Fluorescent Detection of Quercetin

For the measurement of quercetin, different volume of quercetin stock solution (30 mM) was mixed with the as-synthesized GSH-Cu NCs/Al^3+^ solution, and then, the mixed solutions were replenished to 3 mL with PBS solution (0.1 M, pH 7.0). Following incubation at room temperature for 5 min, the fluorescence measurements were performed with excitation at 365 nm.

### 2.4. Application in Real Samples

Tea samples were procured from supermarket and pretreated according to the literature with slight modification [25]. Samples were filtered through a poly (ether sulfone) filter (pore size: 0.22 μm) and subsequently used for preparing the quercetin stock solution, which was primarily different from the protocol detailed in Section 2.3. The feasibility of the designed fluorescent sensor in practical application was verified by standard spiking method. Each specimen was measured three times to determine the relative standard deviation (RSD).

## 3. Results

### 3.1. Characterization of the GSH-Cu NCs/Al^3+^

**Figure 2:**
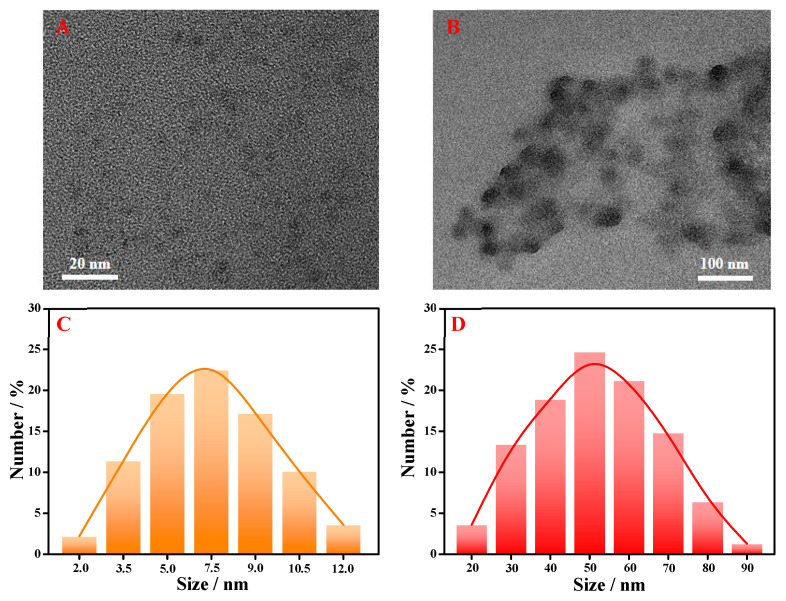
TEM images of the as-synthesized GSH-Cu NCs (**A**) and GSH-Cu NCs/Al^3+^ (**B**). Size-distribution histograms of GSH-Cu NCs without (**C**) and with Al^3+^ ion (**D**).

The formation of fluorescent GSH-Cu NCs mainly included two steps: firstly, GSH molecules reduced Cu(II) of CuSO_4_ to Cu(I) or Cu (0), and then the reduced Cu(I) or Cu (0) coordinated with the -SH groups in GSH molecules to generate a Cu-thiolate complexes-based insoluble colloid; subsequently, the addition of NaOH induced the redispersion of Cu-thiolate complexes, resulting in the stable fluorescent GSH-Cu NCs [26]. The micromorphology of the synthesized nanomaterials was observed via TEM images, and the results are shown in Figure 2. As illustrated, the synthesized GSH-Cu NCs was well dispersed in an aqueous medium with the spherical nanostructure (Figure 2A), and the average particle size measured from TEM image was 7.5 ± 0.5 nm (Figure 2C). After the addition of Al^3+^ ion, the monodisperse GSH-Cu NCs displayed a significant aggregation state with a mean size of 50 ± 5 nm, as in Figure 2B,D. Correspondingly, the prepared GSH-Cu NCs/Al^3+^ solution revealed distinct milky turbidity, while the GSH-Cu NCs solution remained optically transparent, as seen in Figure S1.

**Figure 3:**
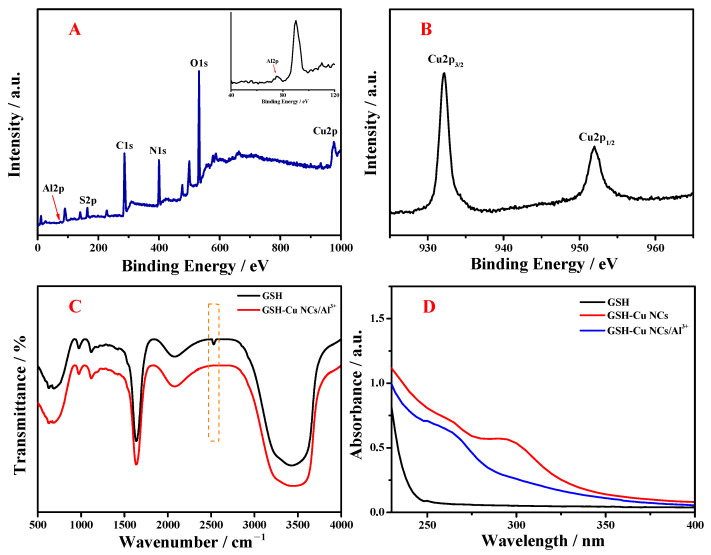
(**A**) Full-scan XPS spectrum of the synthesized GSH-Cu NCs/Al^3+^. Inset: magnified XPS spectrum of Al 2p. (**B**) High-resolution XPS spectrum of the Cu 2p region. (**C**) FT-IR spectra of the pure GSH molecules (black line) and the resultant GSH-Cu NCs/Al^3+^ (red line). (**D**) UV-vis absorption spectra of the GSH molecules (black line), GSH-Cu NCs without (red line) and with Al^3+^ ion (blue line).

The elemental species and valence states were studied using XPS and EDS techniques. As demonstrated in Figure 3A, the as-anticipated elements including C, N, O, S, Cu, and Al were found in the resultant GSH-Cu NCs/Al^3+^. This result was in full agreement with EDS characterization (Figure S2). The high-resolution XPS spectrum of the Cu2p electrons in the GSH-Cu NCs/Al^3+^ was displayed in Figure 3B, in which two binding energy peaks at approximately 932.1 and 952.0 eV were, respectively, assigned to Cu2p3/2 and Cu2p1/2 of Cu atom. Moreover, a satellite peak attributed to divalent copper [Cu(II)] was not observed at 943.0 eV, suggesting that Cu(II) was completely reduced during the preparation of GSH-Cu NCs/Al^3+^. The above results were identical to the previous reports [27,28]. The FT-IR spectra were employed for identifying the surface structure of the resultant GSH-Cu NCs/Al^3+^, using the pure GSH as the reference. As shown in Figure 3C, the characteristic peak of GSH molecules at 2531 cm^−1^, which was ascribed to the S-H stretching vibration, disappeared in the FT-IR spectrum of GSH-Cu NCs/Al^3+^, indicating that GSH molecules as the stabilizing ligands were fixed on the surface of formed Cu NCs via forming a Cu-S bond [29,30]. Figure 3D revealed the UV-vis absorption spectra of GSH (black line), GSH-Cu NCs without (red line) and with Al^3+^ ion (blue line). Compared with the pure GSH, the prepared GSH-Cu NCs possessed a distinct absorption band at 295 nm. Moreover, this band underwent a blue shift to 265 nm upon the addition of Al^3+^ ion. The above characterization demonstrated the successful synthesis of GSH-Cu NCs/Al^3+^.

**Figure 4:**
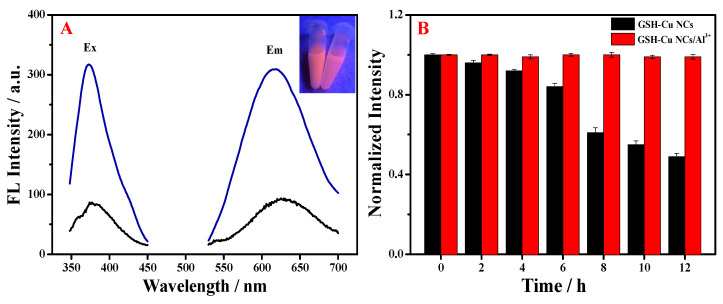
(**A**) Excitation and emission spectra of the fluorescent GSH-Cu NCs without (black line) and with Al^3+^ ion (blue line). Inset: photo of the free GSH-Cu NCs (right) and the resultant GSH-Cu NCs/Al^3+^ solutions (left) under the 365 nm UV irradiation. (**B**) Storage stability of the free GSH-Cu NCs and the resultant GSH-Cu NCs/Al^3+^ solutions under the condition of 4 °C.

The fluorescence properties of the synthesized nanomaterials were further investigated. As displayed in Figure 4A, the emission (Em) wavelength of the fluorescent GSH-Cu NCs (black line) was peaked at 625 nm under an excitation (Ex) wavelength of 380 nm. Following the introduction of Al^3+^ ion (blue line), the fluorescence emission wavelength was blue-shifted to 615 nm, and the fluorescence intensity was remarkably enhanced as well. This optical behavior can be interpreted as an aggregation-induced emission of the resultant GSH-Cu NCs triggered by the addition of Al^3+^ ion, and the Al^3+^-induced aggregation mechanism of the resultant GSH-Cu NCs is ascribed to the high-affinity electrostatic and coordination interactions between the added Al^3+^ ions and the hydroxyl groups in the resultant GSH-Cu NCs [31,32,33]. Under a 365 nm UV light, the GSH-Cu NCs/Al^3+^ solution displayed an intense orange-red fluorescence, which was significantly brighter than that of the free GSH-Cu NCs solution (inset in Figure 4A). Notably, the storage stability of the free GSH-Cu NCs at 4 °C was markedly improved in the presence of Al^3+^ ion (Figure 4B).

### 3.2. Optimization Assay

The experimental parameters would affect the performance of the designed GSH-Cu NCs/Al^3+^-based fluorescent sensor, including the molar ratios between CuSO_4_ and GSH, the added amounts of Al^3+^ ion, salt concentrations (i.e., NaCl), and pH values.

**Figure 5:**
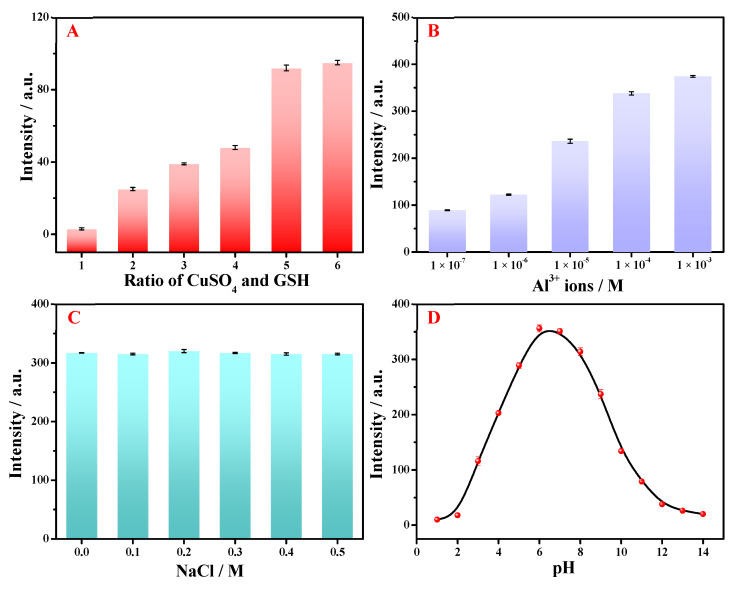
Effects of (**A**) the molar ratio between CuSO_4_ and GSH (1 to 6: 1:1, 1:2, 1:3, 1:4,1:5, 1:6), (**B**) the concentration of Al^3+^ ion on the fluorescent GSH-Cu NCs. (**C**) The stability of the resultant GSH-Cu NCs/Al^3+^ under the condition of high-concentration of NaCl. (**D**) The fluorescence intensity of GSH-Cu NCs/Al^3+^ in full pH range from 1.0 to 14.0.

The effect of molar ratio between CuSO_4_ and GSH on the fluorescent GSH-Cu NCs was primarily investigated, and the results are displayed in Figure 5A. As illustrated, the intensities of fluorescent GSH-Cu NCs were continuously enhanced with the increasing molar ratios between CuSO_4_ and GSH, and hardly enhanced until the molar ratio up to 1:5, so that this value was chosen as the optimal condition. The different concentrations of Al^3+^ ion for the fluorescence enhancement of the resultant GSH-Cu NCs/Al^3+^ was shown in Figure 5B. It can be seen that an enhancement in the intensity of fluorescent GSH-Cu NCs was observed with increasing concentration of Al^3+^ ion. To avoid the potential interference from excessive concentration of Al^3+^ ion in the subsequent detection of quercetin, an optimal Al^3+^ ion addition of 1 × 10^−4^ M was chosen. The stability of the synthesized GSH-Cu NCs/Al^3+^ is an important factor in the practical application, thus, the fluorescence intensities in different concentrations of salt solution were measured. As depicted in Figure 5C, there was no significant variation under 0.5 M NaCl, demonstrating the favorable stability of the resultant GSH-Cu NCs/Al^3+^ in high-salt conditions. In addition, the influence of solution pH on the resultant GSH-Cu NCs/Al^3+^ was investigated. As shown in Figure 5D, the florescence emission was primarily observed within the pH range of 5.0–8.0, and highly acidic or alkaline conditions are unfavorable for the formation of GSH-Cu NCs, resulting in the lower florescence intensity [34]. The maximum fluorescence intensity was approximately at pH 6.0. Considering that the pH values of the obtained tea samples range from 6.0 to 8.0, thus the pH of GSH-Cu NCs/Al^3+^ was adjusted at pH 6.0 for further measurements.

### 3.3. GSH-Cu NCs/Al^3+^ as a Fluorescent Switch-Off Sensor for Quercetin

**Figure 6:**
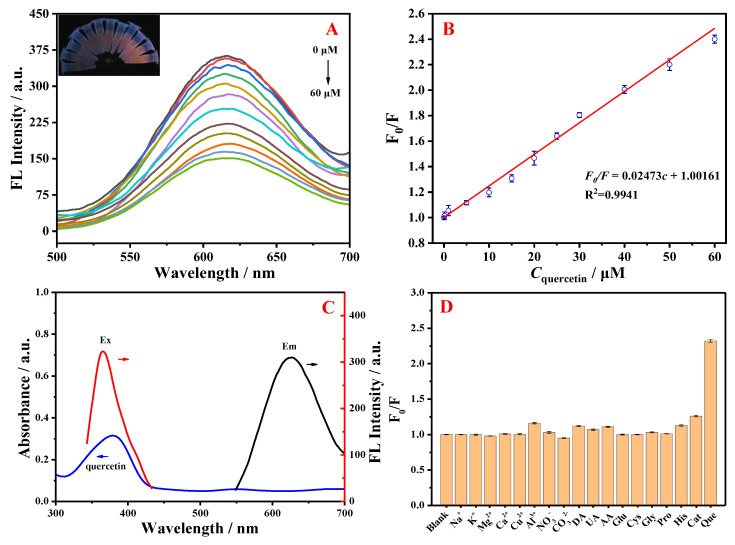
(**A**) Emission spectra of the fluorescent GSH-Cu NCs/Al^3+^ with increasing concentration (0, 0.1, 1, 5, 10, 15, 20, 25, 30, 40, 50 and 60 μM) of quercetin. Inset: photo of the resultant GSH-Cu NCs/Al^3+^ solution with various quercetin concentrations under the 365 nm UV irradiation (the concentration increased from right to left). (**B**) Stern–Volmer linear curve between the value of fluorescence quenching ratios (*F*_0_*/F*) and concentration of quercetin. (**C**) Excitation and emission spectra of the fluorescent GSH-Cu NCs/Al^3+^ overlapping with UV-vis absorption spectrum of quercetin. (**D**) Fluorescence quenching ratios (*F*_0_*/F*) of the GSH-Cu NCs/Al^3+^ in the sample solution containing quercetin (50 μM) and other interferents (100 μM). Que represents the quercetin.

The response of fluorescent GSH-Cu NCs/Al^3+^ for different concentrations of quercetin was evaluated. Considering the importance of reaction time, a measurement was conducted to investigate the effect of this parameter on fluorescence changes (*F*_0_*/F*, where *F*_0_ and *F* represent the intensities of the prepared fluorescent GSH-Cu NCs/Al^3+^ in the absence and presence of the targeted analyte, respectively). As depicted in Figure S3, the fluorescence quenching induced by quercetin (10 μM) tended to equilibrate after incubation with GSH-Cu NCs/Al^3+^ for 2 min. Hence, the optimal incubation time was chosen as 2 min. The fluorescence responses of GSH-Cu NCs/Al^3+^ to varying concentrations of quercetin were systematically quantified. As illustrated in Figure 6, the fluorescence emission of the system exhibited a gradual decrease with increasing quercetin concentration, and correspondingly, the fluorescent GSH-Cu NCs/Al^3+^ solutions containing different amounts of quercetin under the 365 nm UV irradiation became progressively weaker with the escalating amounts of quercetin (inset in Figure 6A). A good linear relationship was acquired between the fluorescence quenching ratios (*F*_0_*/F*) of the resultant GSH-Cu NCs/Al^3+^ and the concentration of quercetin in the range of 0.1–60 μM. The linear equation would be described as *F*_0_*/F* = 0.02473*c* + 1.00161 (*c* represents the quercetin concentration), with a significant correlation coefficient of 0.9941 in Figure 6B. A detection limit for quercetin was calculated to be 24 nM (S/N = 3), which is comparable to or even lower than the detection limits of other established methods and material-based fluorescent sensors for quercetin described in Tables S1 and S2.

According to previous reports, this fluorescence quenching mechanism may be related to the internal filter effect (IFE) [35,36,37]. In this system, the fluorescence spectra of the resultant GSH-Cu NCs/Al^3+^ and UV-vis absorption spectrum of quercetin were investigated for elucidating the mechanism. In Figure 6C, the significant overlap between the absorption spectrum of quercetin and the excitation spectrum of fluorescent GSH-Cu NCs/Al^3+^ demonstrated that the fluorescence quenching of the GSH-Cu NCs/Al^3+^ was relevant to the influence of IFE. Furthermore, the selectivity of GSH-Cu NCs/Al^3+^ towards quercetin was explored to evaluate the performance of the proposed fluorescent sensor. Various potentially co-existing substances, including structural analog [catechin hydrate (Cat)], amino acids [cysteine (Cys), glycine (Gly), proline (Pro)], bioactive small molecules [ascorbic acid (AA), dopamine (DA), glucose (Glu), histidine (His), uric acid (UA)], anions [NO_3_^−^, CO_3_^2−^], metal ions [Na^+^, K^+^, Mg^2+^, Ca^2+^, Cu^2+^, Al^3+^], were selected as the interferents. As revealed in Figure 6D, quercetin could significantly quench the fluorescence emission of the resultant GSH-Cu NCs/Al^3+^, while other interfering substances exhibited negligible responses in the fluorescence sensing system, even at concentrations higher than that of quercetin. The above results demonstrated that the designed GSH-Cu NCs/Al^3+^-based fluorescent sensor has favorable selectivity for the determination of quercetin.

### 3.4. Quercetin Sensing in Real Samples

The feasibility of the proposed fluorescent sensor was assessed through detection of quercetin in food matrices. Commercially available tea beverages (green tea, black tea and oolong tea) were employed as the food matrices and subjected to specific pretreatment procedures. Then, the pretreated samples were spiked with several different concentrations of quercetin (5, 10 and 20 μM) for fluorescence assay. As illustrated in Table 1, the satisfactory recoveries of GSH-Cu NCs/Al^3+^ as the fluorescent sensor for quercetin detection in tea samples were obtained from 96.4% to 108.3% with an intra-day RSD below 4.0% (n = 3), which indicated that the GSH-Cu NCs/Al^3+^-based fluorescent sensor can be served as an effective and reliable approach for sensing quercetin in real samples.

## 4. Conclusions

In summary, water-soluble fluorescent GSH-Cu NCs were synthesized using glutathione as both reductant and protectant through a facile and green one-pot approach. The introduction of Al^3+^ ion significantly enhanced the stability and fluorescence of the resultant GSH-Cu NCs via an aggregation-induced emission mechanism. Interestingly, the fluorescence emission of the as-fabricated GSH-Cu NCs/Al^3+^ could be efficiently quenching in the presence of quercetin. The quenched fluorescence intensity exhibited a good linear relationship with quercetin concentrations in the range from 0.1 μM to 60 μM, and the limit of detection was 24 nM. Moreover, the proposed GSH-Cu NCs-Al^3+^-based fluorescent sensor was applied for quercetin detection in food matrices with satisfactory recoveries ranging from 96.4 to 108.3%. This work may provide an efficient fluorescent strategy for sensing diverse natural flavonoids.

## Figures and Tables

**Table 1:** Determination of quercetin in food matrices.

Samples	Spiked (μM)	Found (μM)	Recovery (%, n = 3)	RSD (%, n = 3)
Green tea	5	5.2	103.5	1.1
	10	10.8	108.3	3.2
	20	21.5	107.7	3.8
Black tea	5	4.9	99.1	2.9
	10	10.4	101.6	2.5
	20	21.0	102.6	3.4
Oolong tea	5	4.9	97.5	2.2
	10	9.8	96.4	3.1
	20	20.6	100.6	2.2

## Data Availability

The original contributions presented in the study are included in the article. Further inquiries can be directed to the corresponding authors.

## Supplementary Materials

The following supporting information can be downloaded at https://www.mdpi.com/article/10.3390/foods14152750/s1. Figure S1: Photograph of the prepared GSH-Cu NCs (right) and GSH-Cu NCs/Al^3+^ (left) solutions under the irradiation of visible light; Figure S2: EDS spectra of the GSH-Cu NCs/Al^3+^; Figure S3: Effect of incubation time on the fluorescence quenching of GSH-Cu NCs/Al^3+^ induced by 10 μM quercetin; Table S1: Comparison of the proposed fluorescence detection method for quercetin with other reported methods [38,39,40,41,42,43]; Table S2: Comparison of the fluorescent sensor based on different nanomaterials for the detection of quercetin [1,5,12,35,36,37,44,45].
