# Supplementary material for: Glutathione-Stabilized Copper Nanoclusters as a Switch-Off Fluorescent Sensor for Sensing of Quercetin in Tea Samples
Source: Foods. 2025 Aug 6;14(15):2750. doi: 10.3390/foods14152750 (PMC12345651; doi:10.3390/foods14152750)
Supplement: Supplementary file 1 [file foods-14-02750-s001.zip › foods-3747267-supplementary.pdf]

Glutathione-Stabilized Copper Nanoclusters as a Switch-off Fluorescent Sensor for Sensing of Quercetin in Tea Samples

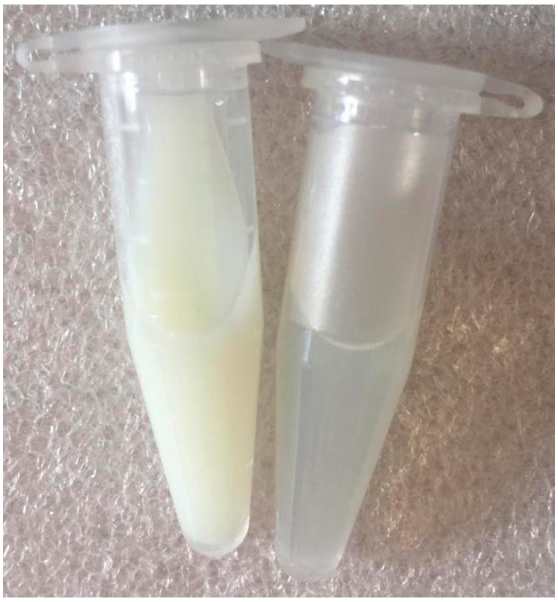

Figure S1. Photograph of the prepared GSH-Cu NCs (right) and GSH-Cu NCs/Al^{3+} (left) solutions under the irradiation of visible light.

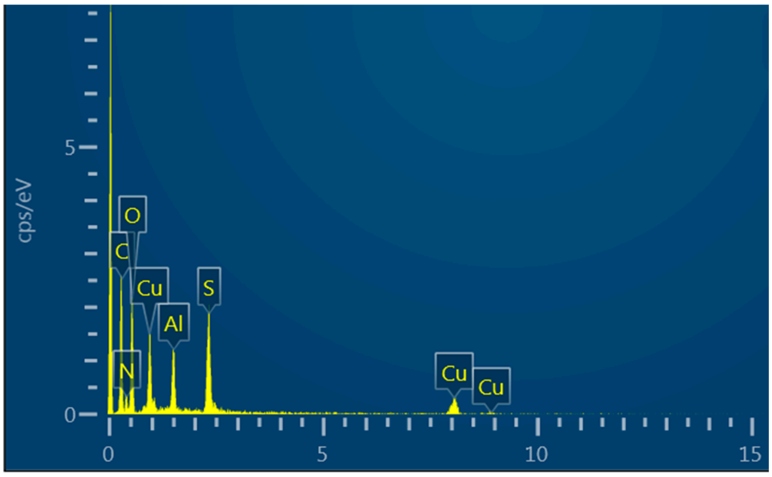

Figure S2. EDS spectra of the GSH-Cu NCs/Al^{3+}.

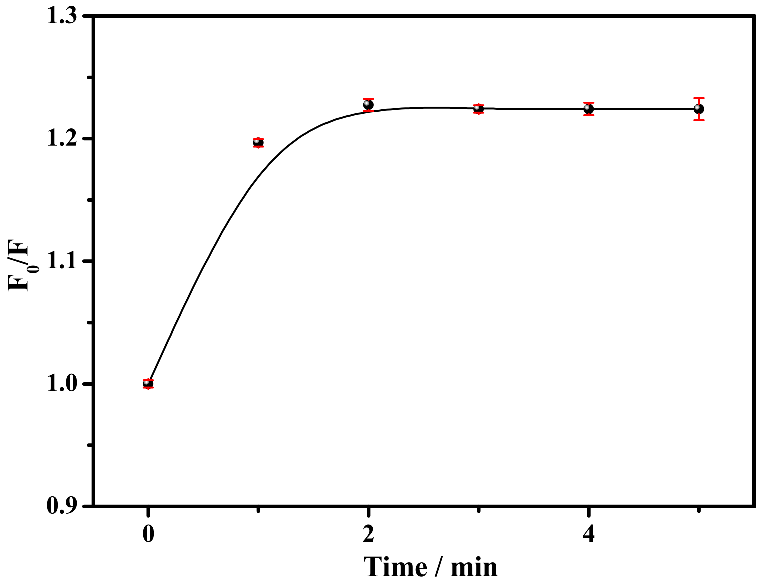

Figure S3. Effect of incubation time on the fluorescence quenching of GSH-Cu NCs/Al^{3+} induced by 10 μM quercetin.

Table S1. Comparison of the proposed fluorescence detection method for quercetin with other reported methods.

**Methods**	**Linear range (μM)**	**LOD (μM)**	**Ref.**
HPLC-PDA	0.52 - 170	0.25	[1]
Capillary electrophoresis-ultraviolet	50 - 500	30	[2]
Molecularly imprinted polymer-based optical sensor	2.5 - 80<br>80 - 200	0.7	[3]
Surface-enhanced Raman scattering	0.33 - 3300	0.076	[4]
Electrochemical	0.001 - 329	0.003	[5]
Ratiometric fluorescent probe	0 - 35	0.09	[6]
**Fluorometry**	**0.1 - 60**	**0.024**	**This work**

Table S2. Comparison of the fluorescent sensor based on different nanomaterials for the detection of quercetin.

Materials	Reaction Time (min)	Linear range (μM)	LOD (nM)	Ref.
UiO-66-TCPP	8	0 - 66, 99 - 230	26	[7]
nano-Zr-MOF	-	0.1 - 70	26	[8]
PEG-SQDs	20	0.1 - 45	14	[9]
PBA-CDs	-	0 - 28.5	28	[10]
PC-Al	2	0 - 50	23	[11]
AA-Cu NCs	1	0.7 - 50	190	[12]
Cys-Cu NCs	-	1 - 20	35	[13]
PEI-Cu NCs	2	0.1 - 90, 110 - 200	72	[14]
**GSH-Cu NCs/Al^{3+}**	**2**	**0.1 - 60**	**24**	**This work**

References

[1] L. Q. Li, J. Cheng, F. Lu, Y. D. Du, Y. Xie, C. Zhou, J. Zhang, Y. H. Feng. Optimized HPLC extraction method of quercetin and berberine based on response surface analysis. RSC Advances, 2023, 13, 29427-29437.

[2] P. Hemwech, A. Obma, S. Detsangiamsak, S. Wirasate, P. Wilairat, R. Chantiwas. Capillary surface modification using millimolar levels of aminosilane reagent for highly efficient separation of phenolic acids and flavonols by capillary electrophoresis with UV detection. Phytochemical Analysis, 2023, 34, 621-631.

[3] T. Di Giulio, I. M. Asif, M. Corsi, S. Rajpal, B. Mizaikoff, N. Ditaranto, G. E. De Benedetto, C. Malitesta, G. Barillaro, E. Mazzotta. A molecularly imprinted polymer-based porous silicon optical sensor for quercetin detection in wines. ACS

Applied Materials & Interfaces, 2025, 17, 12663-12675.

[4] X. Y. Fang, J. L. Ma, C.J. Gu, W. Xiong, T. Jiang. Synchronous enhancement of electromagnetic and chemical effects-induced quantitative adsorptive detection of quercetin based on flexible polymer-silver-ZIF-67 SERS substrate. Sensors and Actuators B: Chemical, 2023, 378, 133176.

[5] V. Mariyappan, N. Karuppusamy, S. M. Chen, P. Raja, R. Ramachandran. Electrochemical determination of quercetin using glassy carbon electrode modified with $WS_2/GdCoO_3$ nanocomposite. Microchimica Acta, 2022, 189, 118.

[6] B. G. Wang, X. Liu, W. M. Duan, S. S. Dai, H. S. Lu. Visual and ratiometric fluorescent determination of Al^{3+} by a red-emission carbon dot-quercetin system. Microchemical Journal, 2020, 156, 104807.

[7] Y. R. Wang, X. L. Sun, Y. F. Zhou, J. C. Liu, H. Y. Zhu, R. Jiang, Y. J. Miao, Y. Q. Fu. A ratiometric fluorescent probe based on UiO-66-TCPP for selective and visual detection of quercetin in food. Food Chemistry, 2024, 457, 140198.

[8] S. T. Feng, F. R. Tang, F. S. Wu, J. Zhang. One-pot synthesis of nano Zr-based metal-organic frameworks for fluorescence determination of quercetin and Hg2+. Food Chemistry, 2024, 432, 137173.

[9] W. J. Jiang, R. He, F. Zhang, L. Wang, Y. L. Wei. Water-soluble sulfur quantum dots as a potential sensitive fluorescent probe for quercetin detection and cell imaging. Food Chemistry, 2025, 464, 141618.

[10] M. Gao, M. He, R. Xing, X. F. Wang, Z. Wang. Borate-modified carbon dots as a probe for quercetin in plants. Analyst, 2021, 146, 590-596.

[11] X.Q. Su, W. W. Sun, L. Wang, X. Zhang, M. X. Liu, Y. Qu, L. J. Ming. A "turn-on" Al(III)-mediated fluorescent micro-probe for quercetin sensing. Sensors and Actuators B: Chemical, 2024, 420, 136497.

[12] Z.F. Cai, H.Y. Li, J.L. Wu, L. Zhu, X.R. Ma, C.F. Zhang. Ascorbic acid stabilised copper nanoclusters as fluorescent sensors for detection of quercetin. RSC Advances, 2020, 10, 8989-8993.

[13] T. Sasikumar, M. Ilanchelian. Water-soluble luminescent copper nanoclusters as a fluorescent quenching probe for the detection of rutin and quercetin based on the

inner filter effect. Luminescence, 2021, 36, 326-335.

[14] S. Zhang, J. H. Li, S. Y. Huang, X. R. Ma, C. F. Zhang. Novel blue-emitting probes of polyethyleneimine-capped copper nanoclusters for fluorescence detection of quercetin. Chemical Papers, 2021, 75, 3761-3769.
